# What Are the Indications for Extraction?

**Published:** 1869-07

**Authors:** C. W. Stanley


### WHAT ARE THE INDICATIONS FOR EXTRACTION?

BY C. W. STANLEY, D. D. S.

[Read before the Indiana Dental Association.]

I do not propose to occupy the attention of this Association for any considerable length of time, but shall try and furnish a few thoughts which may serve to open the discussion on this subject.

So long as there remains so wide a difference in the qualifications of the practitioners of Dentistry, and in their ability to successfully treat the diseases of the teeth, there will be a wide difference in their appreciation of their value, and the indications to them for or against extraction will be governed both by their ability and their appreciation. The Dentist who is able to restore the teeth to health and usefulness for years, and often through life, after disease and decay has attacked them, will appreciate their value and be slow to advise their removal for ordinary causes. While he who feels his inability to meet the requirements in the case, and baffled by his imperfect attempts to save them, which generally result in their extraction and replacement by artificial substitutes, with increased pain and expense, will lose his appreciation of their value and see indications for extraction in the mildest forms of disease. Hence, we see these indications do not imply any particular pathological condition of the teeth or their surroundings, but are on a sliding scale and are presented to each one in exact accordance with the position which we occupy relatively in the profession.

With a certain class of persons (I will not insult you by calling them Dentists,) the simple privilege to extract a tooth is sufficient indication for its removal, especially if there is an opportunity to replace it with an artificial one. These have neither the fear of God or of the law before them, and professionally are so low that the light never reaches them, and the light they do get in regard to the indications requiring extraction accords with the position which they occupy *to* the profession. Mark, I do not say *in* the the profession.

Another thinks his wares so desirable that he considers slight decay of the teeth, inflammation of the gums, or some slight disease of the surrounding parts sufficient cause for their removal, and I have known such, after having removed the enamel from a sound tooth by filing, for the purpose of filling an adjacent one, to recommend extraction as a remedy for the external sensitiveness which resulted, and which they were unable to control. Doubtless these failures in restoring the parts to a normal condition and to preserve the natural teeth for any considerable length of time depreciates their value very decidedly, when compared with artificial substitutes.

Another makes an effort to save the natural organs so long as the requirements are simply mechanical, but quits the field in disgust upon the appearance of pathological conditions, and recommends removing the teeth for extensive decay, with inflammation of the dentine, for inflamed or exposed nerve, for periostitus and for abscess, however slight. Others, and I am happy to believe their numbers are steadily increasing, do not think that these diseases, or any of them, are necessarily indications for extraction, who think the duty of the Dentist is not to mar and mutilate, but to retain as far as possible the form, features and expression of the human face, and this can only be done by using every effort to save these beautiful organs which a benificent God has adapted so completely to the human wants, and which, in their health and beauty are so much superior to any thing artificial, as to bear scarcely any comparison whatever. My sympathies are all with the conservatives in practice, and my aim, as a rule, to extract no teeth which, by my utmost endeavors, I can save comfortable and useful for any considerable length of time. This rule can not always be lived up to, since some of our patients have neither the time or means necessary to justify us in undertaking their cases, and yet those who have not tried it would be surprised at the sacrifice in this way, which even those in moderate circumstances will undergo when fully satisfied of their ability to save their teeth. And not only would I advise saving the teeth which have the crowns remaining, and those with a portion of it remaining, but many roots may, when it is not desirable to replace them with artificial substitutes, be filled with decided advantage. Particularly is this the case with the roots of the bicuspids, both above and below, and those of the inferior molars.

Roots that are broken off below the gum, that have abscess attached to them, or that in other ways are cause of discomfort to the patient, I would extract. Abscess is often an indication for extraction, and yet when attached to a tooth otherwise susceptible of restoration to usefulness, I would ordinarily be governed not so much by the extent to which the surrounding parts were involved as by the amount of time the patient could devote to the treatment.

Patient perseverance, even in difficult cases will, if properly directed, nearly always bring its reward. Teeth rendered useless and painful by absorption of the alveolus, either from old age, from accretions of tartar, or from having lost their antagonists, I would consider fit subjects for the forceps. Hypertrophy of the *cementum*, (exostosis) when producing pain, I would consider cause for extraction. Again, in preparing mouths for artificial work, the question of the relative value comes up and teeth may be condemned, which, under other circumstances, it would be proper to retain, for, in those of inferior quality, which have lost their antagonists and are showing decided disposition to become loose in their sockets.

Extracting sound teeth from a healthy mouth for the purpose of replacing with others, is rarely, in my estimation, justifiable, even where there are but one or two in a place. It is sometimes necessary to remove good teeth for the purpose of correcting irregularity, and sometimes there is so much projection of the anterior ones as to render them not only a deformity, but useless for the purpose for which they were intended, in which case it would be in perfect accordance with correct practice to remove and replace with others.

There may be, and doubtless are, other conditions which would occasionally justify extraction, but I have spoken of the ones which most often occur in my own practice, and will not detain you farther.

